# The effectiveness of ursolic acid niosomes with chitosan coating for prevention of liver damage in mice induced by n-nitrosodiethylamine

**DOI:** 10.1038/s41598-022-26085-2

**Published:** 2022-12-10

**Authors:** Andang Miatmoko, Amelia Anneke Faradisa, Achmad Aziz Jauhari, Berlian Sarasitha Hariawan, Devy Maulidya Cahyani, Hani Plumeriastuti, Retno Sari, Esti Hendradi

**Affiliations:** 1 Department of Pharmaceutical Sciences, Faculty of Pharmacy, Universitas Airlangga, Surabaya, 60115 Indonesia; 2 Stem Cell Research and Development Center, Universitas Airlangga, Surabaya, 60115 Indonesia; 3 Master Program of Pharmaceutical Sciences, Faculty of Pharmacy, Universitas Airlangga, Surabaya, 60115 Indonesia; 4 Department of Veterinary Science, Faculty of Veterinary Medicine, Universitas Airlangga, Surabaya, 60115 Indonesia

**Keywords:** Medical research, Nanoscience and technology

## Abstract

Ursolic acid (UA) is a pentacyclic triterpene carboxylic acid which produces various effects, including anti-cancer, hepatoprotective, antioxidant and anti-inflammatory. However, UA demonstrates poor water solubility and permeability. Niosomes have been reported to improve the bioavailability of low water-soluble drugs. This study aimed to investigate the protective action of UA-niosomes with chitosan layers against liver damage induced by N-Nitrosodiethylamine (NDEA). UA niosomes were prepared using a thin layer hydration method, with chitosan being added by vortexing the mixtures. For the induction of liver damage, the mice were administered NDEA intraperitoneally (25 mg/kgBW). They were given niosomes orally (11 mg UA/kgBW) seven and three days prior to NDEA induction and subsequently once a week with NDEA induction for four weeks. The results showed that chitosan layers increased the particle sizes, PDI, and ζ-potentials of UA niosomes. UA niosomes with chitosan coating reduced the SGOT and SGPT level. The histopathological evaluation of liver tissue showed an improvement with reduced bile duct inflammation and decreasing pleomorphism and enlargement of hepatocyte cell nuclei in UA niosomes with the chitosan coating treated group. It can be concluded that UA niosomes with chitosan coating improved the efficacy of preventive UA therapy in liver-damaged mice induced with NDEA.

## Introduction

Liver damage is the leading global cause of death. In 2017, 1.32 million deaths worldwide or 2–4% of the annual total were due to liver cirrhosis^1,2^. Chemically-induced liver damage results from the metabolic transformation of chemicals into reactive intermediate compounds with the potential to change the structure and function of cellular macromolecules^3^. There are several causes of liver damage, one being exposure to carcinogenic chemicals such as N-nitrosodiethylamine (NDEA) which produces reactive oxygen species (ROS) causing oxidative stress and cellular destruction^4^. Reactive products and free radicals cause an increase in the serum index of liver function such as alanine transaminase (ALT) or serum glutamic-pyruvic transaminase (SGPT), aspartate aminotransferase (AST) or serum glutamic-oxaloacetic transaminase (SGPT), alkaline phosphatase (ALP), gamma-glutamyl transferase (GGT), and total bilirubin. In cases of severe histopathological lesions they cause neoplastic transformation^5^.

UA, a natural pentacyclic triterpenoid compound, has various pharmacological properties including anticancer, hepatoprotective, anti-angiogenesis, apoptosis induction, antioxidant and anti-inflammatory^6,7^. As an antioxidant, UA reduces oxidative stress, modulates the Receptor for Advanced Glycation End Products (RAGE) and decreases NADPH oxidase to prevent the formation of ROS^8^. UA also produces a hepatoprotective effect by maintaining the structural integrity of the liver, reducing high levels of bilirubin, stabilizing serum protein concentrations, and suppressing oxidative stress, inflammation, and apoptosis in the liver^9,10^. Oral administration of a 500 mg/kgBW dose of UA to subjects resulted in a reduction in SGOT and SGPT as well as improvement in liver histopathology^11^.

However, limitations on the oral use of UA, which belongs to class IV Biopharmaceutics Classification System (BCS)^12^, result from poor solubility and absorption. An effective drug delivery system is required to increase its solubility and dissolution. Niosomes represent a vesicular bilayer system composed of non-ionic surfactants and cholesterol in the aqueous phase which can increase drug half-life, enhance stability, and deliver drugs to target organs in a controlled release^13^.

Chitosan, a natural polysaccharide, is a product of alkaline deacetylation of chitin^14^ derived from the exoskeleton of crustaceans^15^ and is widely employed because of its intrinsic polycation properties, low toxicity, and excellent biocompatibility. Modification of UA liposomes with chitosan coating can increase bioavailability, slow drug release in tumor tissue and reduce both dose and side effects. Chitosan can open the tight junctions of epithelial cells, thereby enabling a drug to pass easily through the epithelial membrane via the paracellular pathway^15^. Chitosan also possesses mucoadhesive properties as a result of ionic interactions between positively charged amino groups and negatively charged functional groups on the surface of epithelial cells provide a controlled release while also enhancing absorption in the gastrointestinal tract and intestinal permeability^16^. Therefore, it is expected that the modification of chitosan on the niosomal surface will enhance absorption in the gastrointestinal tract, promote UA niosome accumulation in the liver and increase bioavailability.

In our previous study, optimization of the UA niosome formula found the optimum physical stability in the span 60-cholesterol-UA formula with a mol percent ratio of 3:2:10^17^. Characterization of UA reported that the presence of chitosan showed an increase in the physical stability of UA niosomes. Chitosan coating on UA niosomes affects their physicochemical properties which, in turn, causes an increase in particle size and a more positive zeta potential. Biodistribution evaluation with coumarin-6 labeling revealed that high fluorescence intensity of coumarin-6 indicates high levels of UA in plasma and liver, together with an increase in bioavailability.

In this study, the evaluation of the effectiveness of UA niosomes with chitosan coating as an orally administered in vivo therapy for the prevention of liver damage in NDEA-induced subjects was by means of serum levels of SGOT, SGPT, and liver tissue histopathology.

## Results

### Physical characteristics of UA niosomes

**Figure 1:**
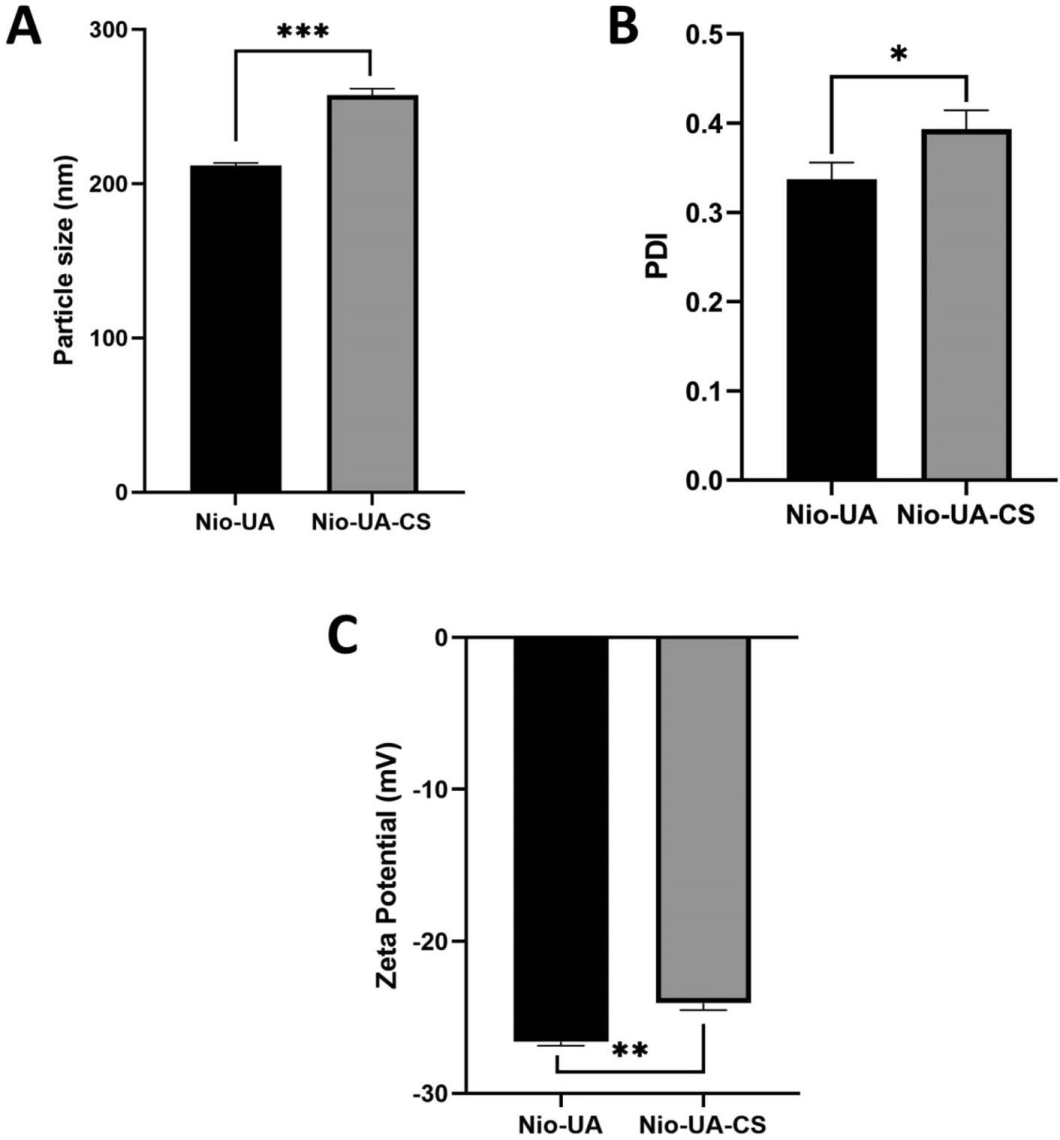
Average (**A**) particle size, (**B**) polydispersity index, (**C**) ζ -potential of Nio-UA and Nio-UA-CS. *p < 0.05; **p < 0.01; ***p < 0.001.

Characteristic UA niosomes parameters include particle size, polydispersity index, and ζ–potential. Measurements were taken from Nio-UA and Nio-UA-CS preparations. A graph of the characteristics of AU niosomes can be seen in Fig. 1A–C.

UA niosomes with chitosan coating (Nio-UA-CS) experienced an increase in particle size from 211.7 ± 1.7 nm (Nio-UA) to 257.4 ± 4.3 nm. A significant difference also occurred in the PDI parameters where the presence of chitosan coating increased the PDI from 0.337 ± 0.018 to 0.393 ± 0.021. The ζ-potential parameter of chitosan coating can also alter the charge from UA niosomes which was initially − 26.6 ± 0.2 mV to − 24.1 ± 0.4 mV. Based on a statistical analysis of the Independent T-Test conducted, the results were p < 0.001 on the particle size parameter, p = 0.03 on the PDI parameter, and p = 0.001 on the ζ-potential parameter, all three of which indicated a significant difference between Nio-UA and Nio.-UA-CS.

### Evaluation of mice body weight

**Figure 2:**
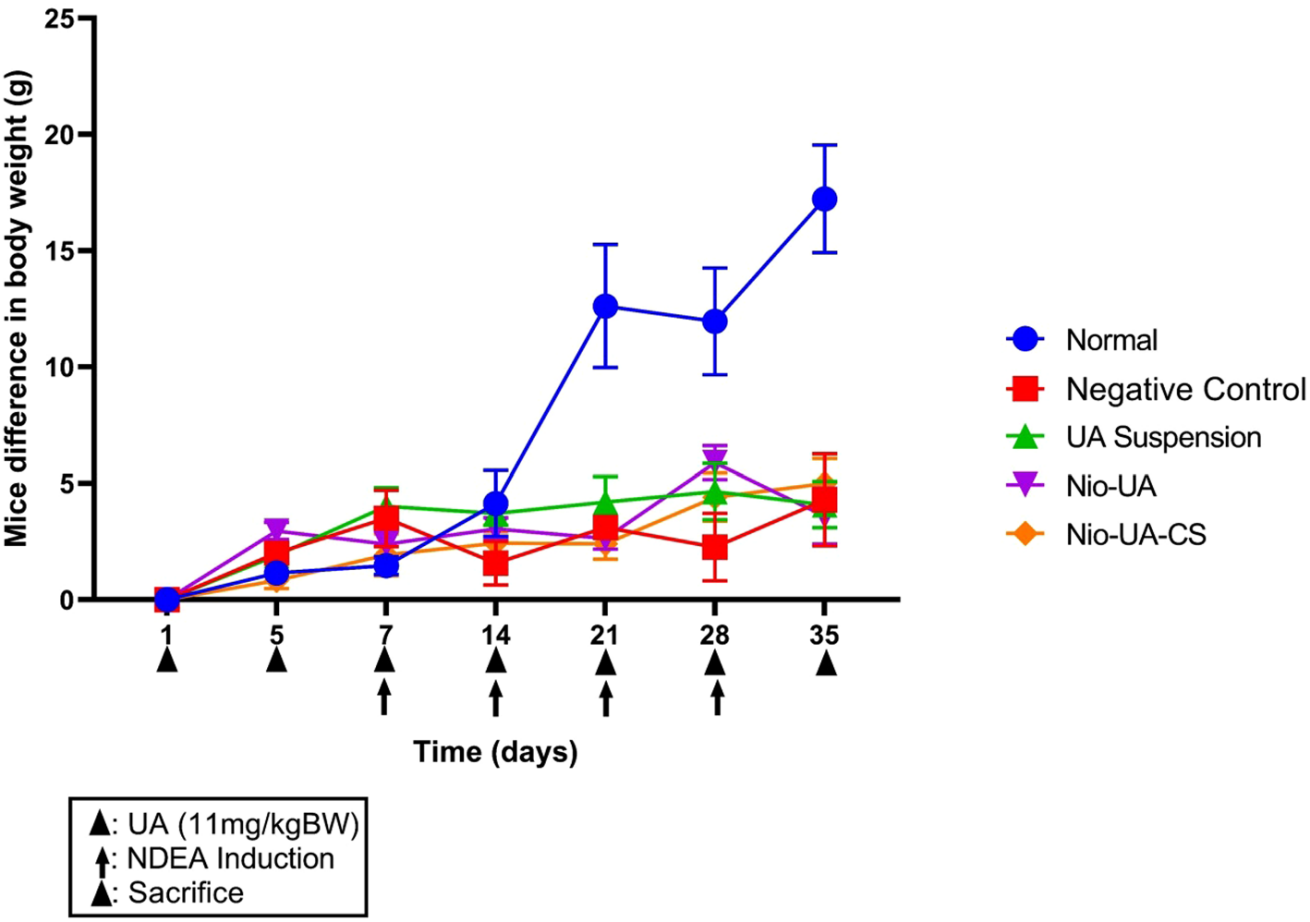
The average difference in body weight of subjects that were treated orally six times with the equivalent of 11 mg UA/kgBW simultaneously with NDEA intraperitoneal induction four times at a dose of 25 mg NDEA/kgBW after which they were sacrificed.

The weight of the subjects in the five groups was recorded every week prior to treatment commencing. The average differences in their weight gain and loss can be seen in Fig. 2.

The body weight profiles of the normal group subjects that had not been induced by NDEA were compared with those of the other four groups that were subjected to NDEA induction on four occasions. The normal group subjects were observed to have experienced the most significant weight gain, while those in the negative control group that had been administered NDEA, but did not undergo UA treatment, demonstrated the smallest difference in body weight. Previous studies of liver inflammation using an NDEA-induced subject model also yielded a weight loss profile^18^. NDEA metabolism in the liver can produce ROS that induce oxidative stress resulting in DNA damage^33^.

### Morphology and organ weight of mice induced with NDEA after administration of UA niosomes

**Figure 3:**
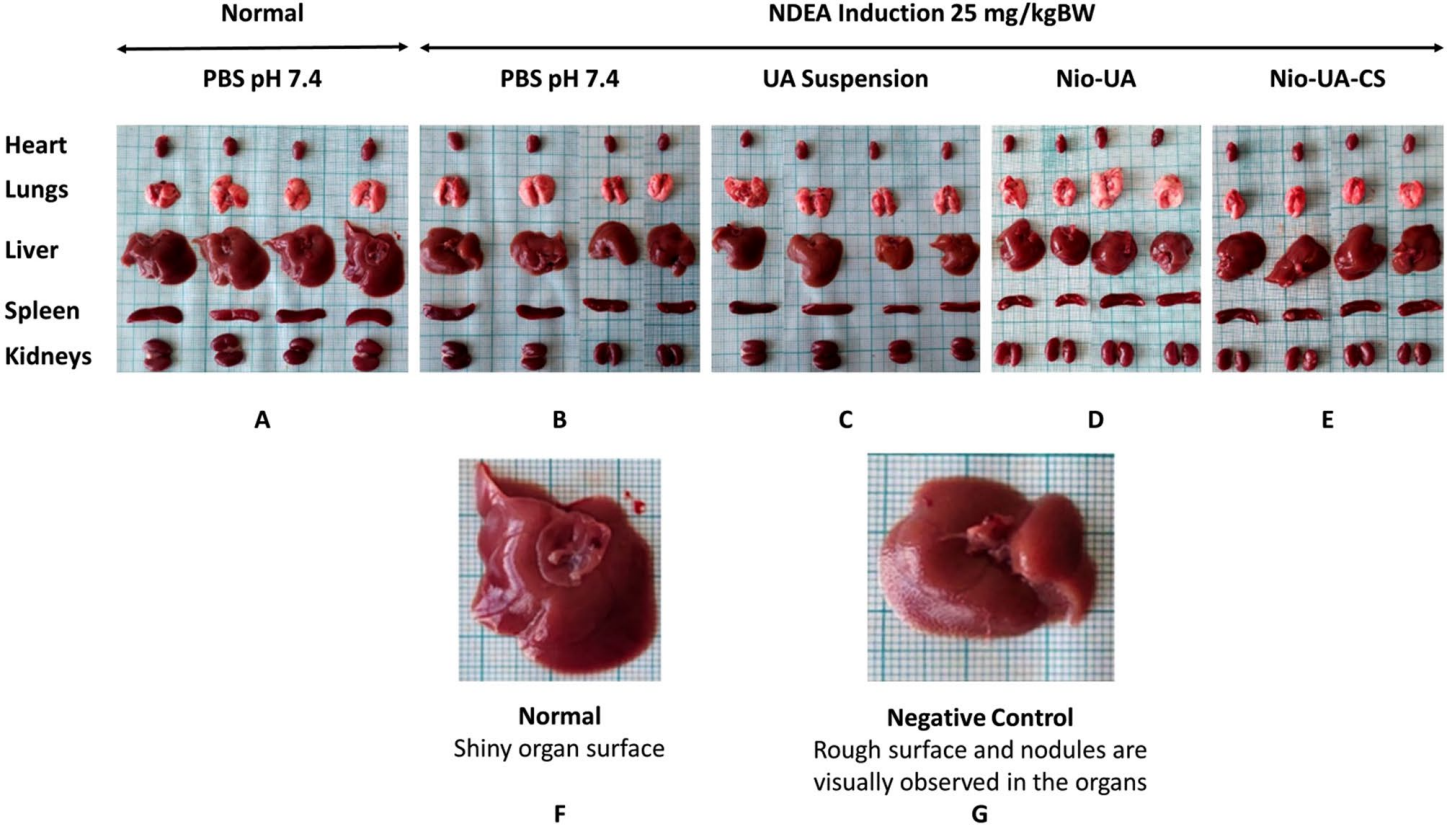
Morphology of the heart, lungs, liver, spleen, and kidneys in group (**A**) of normal subjects with PBS pH 7.4 and oral administration; (**B**) intraperitoneal-induced negative control 25 mg NDEA/kgBW with PBS pH 7.4; induced ip 25 mg NDEA /kgBW with (**C**) UA suspension (**D**) Nio-UA (**E**) Nio-UA-CS which is equivalent to 11 mg UA/kgBW. Differences in liver morphology in the (**F**) normal and (**G**) negative control groups induced by NDEA at a dose of 25 mg/kgBW.

Each organ was photographed post-surgery to determine the qualitative comparison of the morphological organs of subjects in the normal group, the negative control group, the group that received UA, Nio-UA, and Nio-UA-CS suspension treatment. Pictures of complete organs of the normal group subjects, the negative control group subjects induced by NDEA, and the group subjects that received the suspension treatment of UA, Nio-UA, and Nio-UA-CS can be seen in Fig. 3A–G. As it can be seen in Fig. 3A–E, qualitative organ observations confirmed differences in the organs of normal subjects and those which had undergone NDEA induction. In the normal group, the liver surface was bright red and shiny in appearance. Meanwhile, in the negative control group induced by NDEA, a slight color change occurred and several nodules were visible on the surface of the liver, as presented in Fig. 3F,G. This indicates that a 4-week period of NDEA induction damages liver cells.

**Figure 4:**
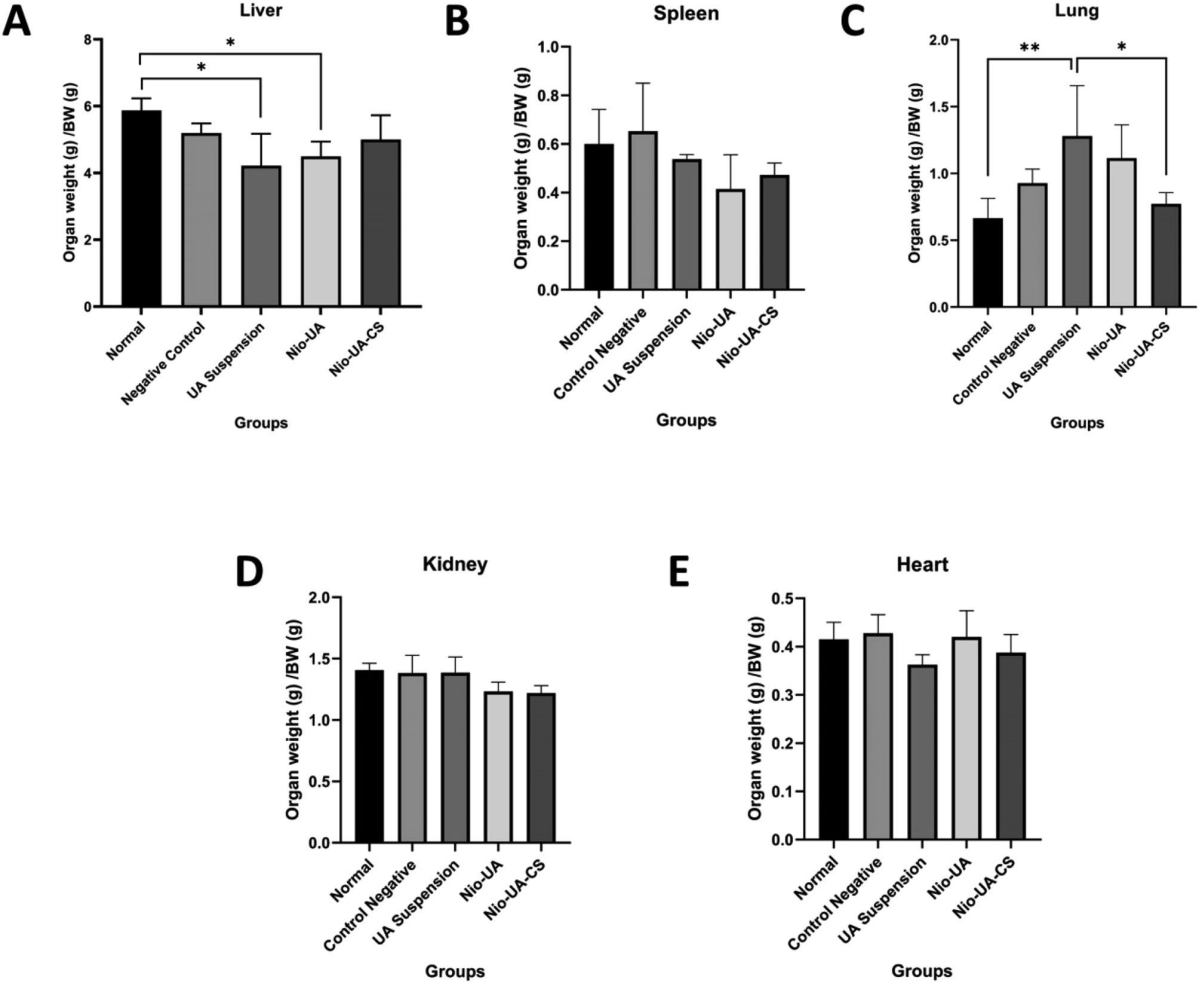
Graph of the relative weight of organs (**A**) liver, (**B**) spleen, (**C**) lungs, (**D**) kidney, (**E**) heart in the normal group and the group which had been NDEA induced with a dose of 25 mg/kgBW and UA suspension treatment, Nio -UA, and Nio-UA-CS which is equivalent to 11 mg UA/kgBW. *p < 0.05; **p < 0.01; ***p < 0.001.

Quantitatively, all the organs of each subject were weighed with each group members’ results being subsequently compared to determine if there was a significant difference. Data on the absolute and relative weight of each organ post-UA treatment and total NDEA induction for 28 days can be seen in Fig. 4A–E. The results show that there were significant differences between groups in the normal group compared to the UA suspension and Nio-UA with regard to the liver and the UA suspension group compared to normal and Nio-UA-CS groups for the lungs.

### Evaluation of SGOT-SGPT levels of mice induced with NDEA after administration of UA niosomes

**Figure 5:**
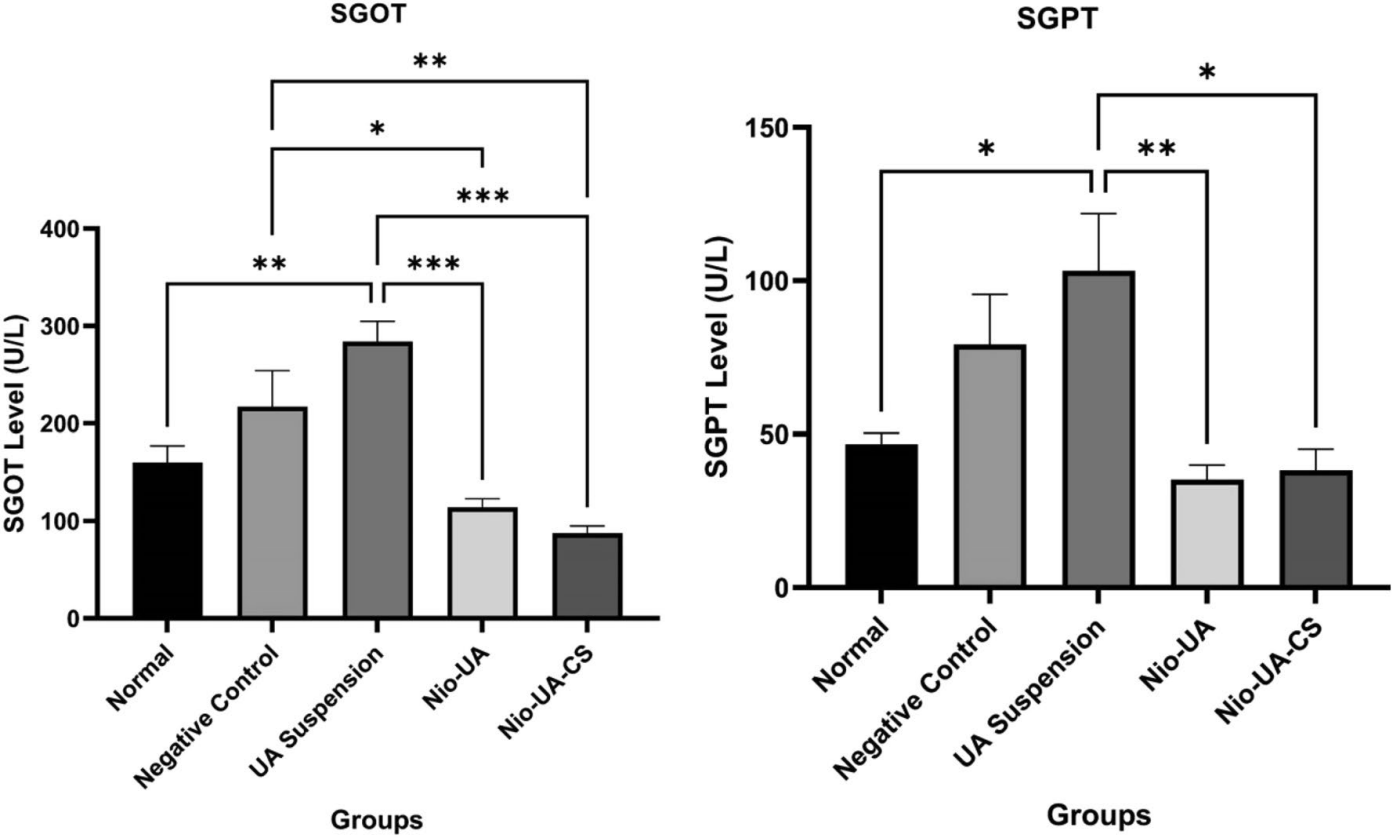
Graph of the average SGOT and SGPT levels in the normal group and the NDEA-induced group at a dose of 25 mg/kgBW with suspension UA, Nio-UA, and Nio-UA-CS treatments which were equivalent to 11 mg UA/kgBW. The data displayed is the mean ± SD (n = 4).

The results of measuring the levels of SGOT and SGPT in the blood serum of subjects in the normal group, negative control, UA suspension, Niosom UA (Nio-UA), and Niosom UA with chitosan coating (Nio-UA-CS) can be seen in Fig. 5. Based on these results, the administration of Nio-UA and Nio-UA-CS can be seen to restore relatively normal serum SGOT and SGPT levels.

### Histopathology evaluation of liver and spleen mice induced with NDEA after administration of UA niosomes

**Figure 6:**
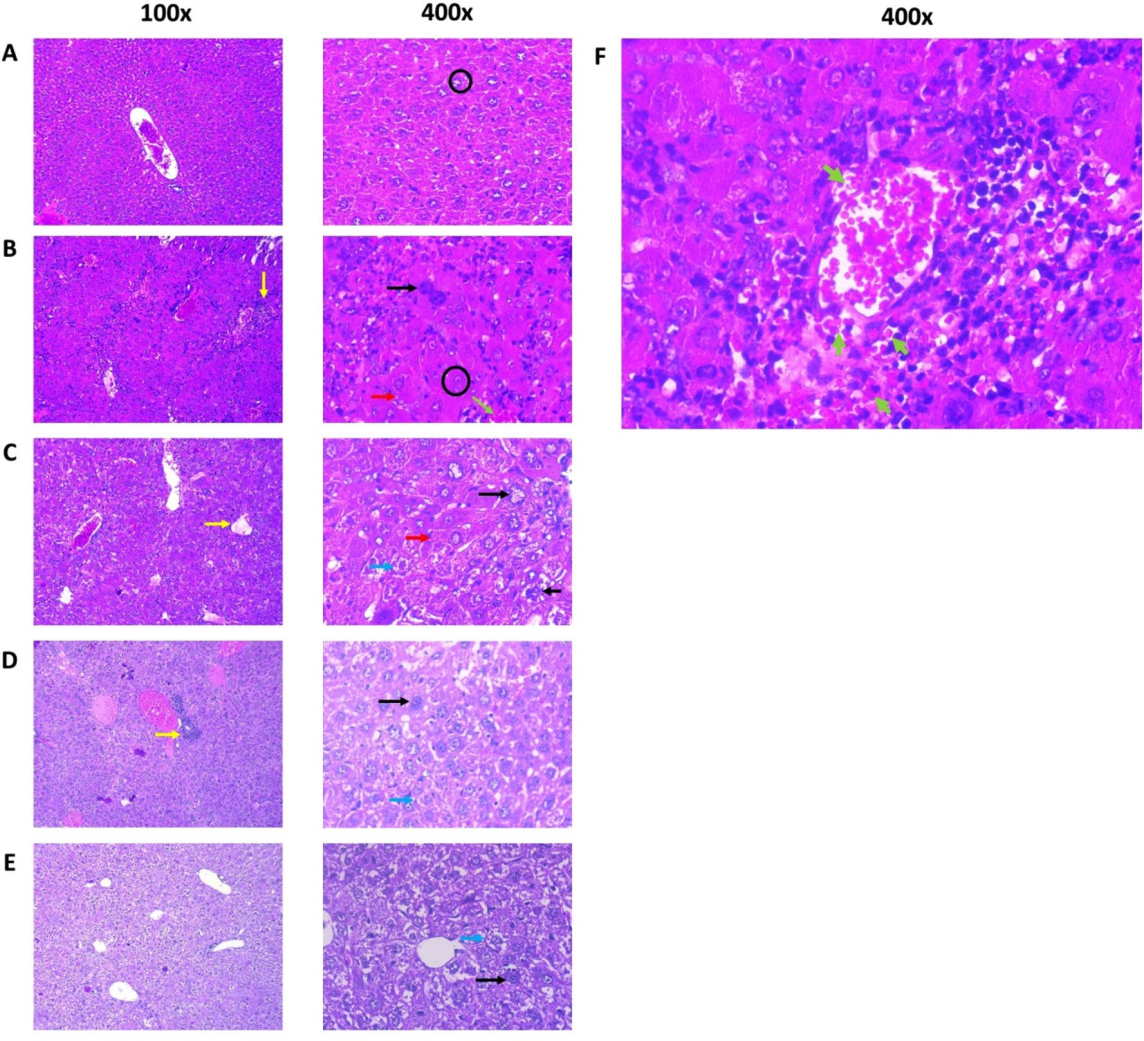
Histopathological picture of subjects’ livers (**A**) Normal, (**B**) Negative control induced with 25 mg NDEA /kgBW ip; (**C**) UA suspension, (**D**) Nio-UA, (**E**) Nio-UA-CS at an equivalent dose of 11 mg UA/kgBW. Picture (**F**) shows the bleeding in the liver tissue of the Negative control group. Image magnification are 100 × and 400 × with H&E staining. Black circle = hepatic plate, black arrow = hyperchromatin and enlarged cell nucleus, yellow arrow = neutrophil infiltration, blue arrow = hydropic degeneration, red arrow = cytoplasmic eosinophilic granules, green arrow = hemorrhage.

The results of microscope observation of liver tissue can be seen in Fig. 6. In this study, in order to further develop the effectiveness of UA niosomes with or without chitosan coating, histopathological analysis of liver and spleen tissue was carried out. Prior to observations being conducted, the tissue was stained with H&E to turn the extracellular matrix and cytoplasm pink, while the cell nucleus was highlighted in blue. The results of observations of subjects’ liver tissue preparations can be seen in Table 1.

**Table 1:** Observation of histopathological liver preparations of subjects in the normal group, negative control, suspension of UA, Nio-UA, and Nio-UA-CS equivalent to a dose of 11 mg UA/kgBW.

Group	Parameter			
	Lobulation	Hemorrhage	Neutrophil infiltration	Dysplastic hepatocytes
Normal	Normal (approximately 40% experience mild degeneration/cloudy swelling)	Negative	Negative (approximately 40% present symptoms of mild port hepatitis)	Negative
Negative control	Enlargement of the hepatocellular plate Hepatic plate not clear Hepatocytes with severe hydropic degeneration (ballooning degeneration)	Mild to moderate around the central vein	Moderate porta hepatitis Several microabscess foci Giant cells	Visible enlargement and size of the nucleus varies and hyperchromatic nuclei Eosinophilic granule cytoplasm Proliferation of biliary duct epithelium
UA suspension	Enlargement of the hepatocellular plate Hepatic plate not clear Hepatocytes with moderate to severe hydropic degeneration Necrotic biliary ducts epithelium	Negative	Mild portal hepatitis was diagnosed (33%) intralobular neutrophil infiltration (50%)	Visible hepatocyte nucleus enlargement Eosinophilic granule cytoplasm Proliferation of biliary duct epithelium (17%)
Nio-UA	Normal liver architecture remains recognizable Mild-severe hydropic degeneration	Negative	Neutrophil infiltration around the bile ducts (pericholangitis)	Cells with hyperchromatic nuclei are observed
Nio-UA-CS	Normal liver architecture remains recognizable Hepatocytes with severe hydropic degeneration	Negative	Mild infiltration of the bile ducts (many are normal)	Several cells with large hyperchromatic nuclei were observed

Parameters observed in this liver tissue include lobulation, bleeding, neutrophil infiltration and dysplastic hepatocytes. Figure 6A, which relates to a normal group, contains normal lobules with normal hepatic plate, uniform cell nucleus size and normal chromatin distribution. No bleeding, neutrophil infiltration and dysplastic hepatocytes were detected. In Fig. 6B, the negative control experienced significant inflammatory cell infiltration, unclear hepatic plate, and erythrocytes outside the blood vessels which is a symptom of bleeding (green arrow). Moreover, pleomorphic nuclei and hyperchromatin, which are indicative of cancer cells, are present indicating that this group is at the initiation stage because the other cell nuclei remain normal. In Fig. 6C, the NDEA group induced with UA suspension treatment presented more portal veins, while darker nuclei thought to be due to necrosis, no proliferation of cells, swelling of cells, enlarged cell nuclei and cytoplasmic eosinophil granules, were indicative of it still being in the initiation phase. In Fig. 6D, the NDEA-induced group subjected to Nio-UA treatment was found to have normal recognizable liver architecture, while in some preparations hyperchromatin nuclei were observed, inflammation occurred around the bile ducts and hepatocyte degeneration ensued (ballooning degeneration). From Fig. 6E, containing the NDEA-induced group with Nio-UA-CS treatment, normal liver architecture can clearly be recognized, several hyperchromatin nuclei, mild inflammation/neutrophil infiltration in the bile ducts, and hepatocyte degeneration (ballooning degeneration) can be observed.

**Figure 7:**
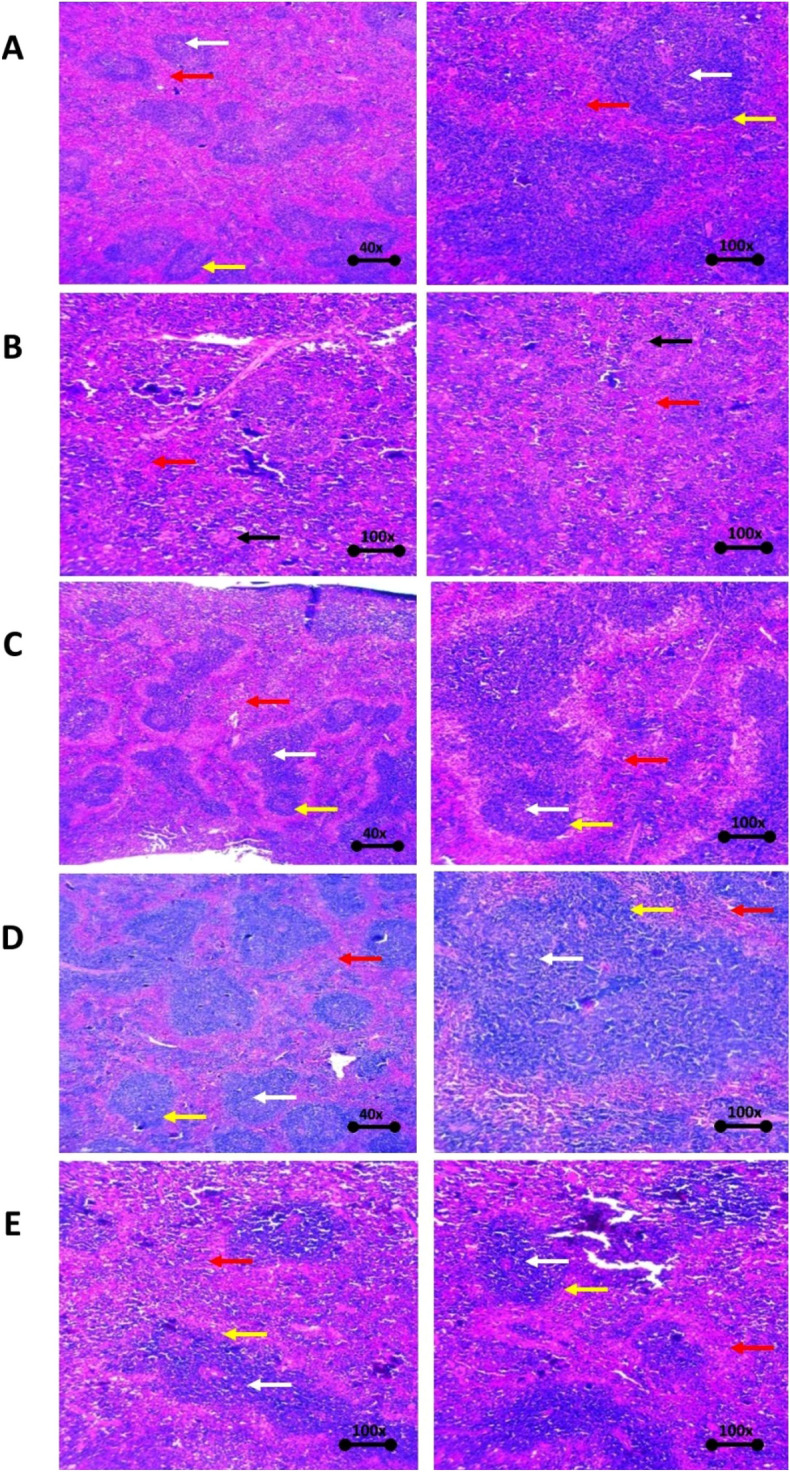
Histopathological picture of the spleen of mice (**A**) Normal, (**B**) Negative control induced with 25 mg NDEA/kgBW ip; (**C**) UA suspension, (**D**) Nio-UA, (**E**) Nio-UA-CS with an equivalent dose of 11 mg UA/kgBW with H&E staining. Red arrow = red pulp, white arrow = white pulp/germinal center, yellow arrow = marginal zone, black arrow = giant cell macrophage.

The comparative observation results relating to spleen tissue viewed through a microscope of the normal group, the negative control group, suspensions of AU, Nio-UA, and Nio-UA-CS can be seen in Fig. 7. The observation results of spleen tissue preparations of the subjects can be seen in Table 2. The parameters observed in the spleen tissue include density, germinal center or white pulp, neutrophil infiltration, and trabeculae. In the normal group (Fig. 7A), under normal density conditions, the white pulp was clearly demarcated with red pulp, normal germinal centers and trabeculae and no neutrophil infiltration. In the negative control group (Fig. 7B), while a decrease in the number of follicles, but no germinal center, was observable, there was an increase in macrophages (giant cells). However, the continued absence of hyperplasia obviated significant damage to the spleen caused by NDEA induction. In group induced by NDEA with UA suspension treatment (Fig. 7C), an increase in the number of germinal centers and marginal proliferation of white pulp lymphoid occurred, indicating the possibility of activation in lymphoid tissue. In group induced by NDEA with Nio-UA treatment (Fig. 7D), a proliferation of white pulp lymphoid tissue was observed, indicating the additional possibility of activation in lymphoid tissue. In group induced by NDEA with Nio-UA-CS treatment (Fig. 7E), mild neutrophil infiltration, marginal proliferation of white pulp lymphoid and an increase in the number of germinal centers was observed indicating the possibility of lymphoid tissue activation.

**Table 2:** Observations of spleen histopathological preparations of mice in the normal group, negative control, UA suspension, Nio-UA, and Nio-UA-CS equivalent to a dose of 11 mg UA/kgBW.

Group	Parameter			
	Density	White pulp/germinal center	Neutrophil infiltration	Trabecular
Normal	Normal	Normal	Negative	Normal
Negative control	Lymphoid tissue appears rather loose	Slight to no visible germinal center, observable increase in macrophages (giant cells)	Negative	Normal
UA suspension	Lymphoid tissue appears rather loose	Marginal proliferation of white pulp lymphoid, increased number of germinal centers	Negative	Normal
Nio-UA	Normal	Marginal proliferation of white pulp lymphoid, a dramatic increase in the number of germinal centers	Mild neutrophil infiltration	Normal
Nio-UA-CS	Lymphoid tissue appears rather loose	Marginal proliferation of white pulp lymphoid, significant increase in the number of germinal centers	Negative	Normal

## Discussion

The increase in particle size of chitosan-coated UA niosomes was due to the fact that chitosan had formed a hydrophilic shell on the niosomal surface through electrostatic interaction^15,19^. Although the particle size increased, coating chitosan on UA niosomes can enhance its effectiveness. It is estimated that, in the presence of chitosan, drug transport can be effected through two pathways, namely; direct cell membranes and paracellular pathways^15^. However, with the addition of chitosan, the value of the polydispersity index (PDI) also increased. The homogeneity criteria for samples with lipid-based carriers was that of PDI < 0.3^20^. The PDI value of Nio-UA remained approximately 0.3 which indicated a relatively homogeneous size distribution. However, chitosan coating significantly increased the PDI value possibly due to the addition of chitosan forming a polymer layer on the surface of the random vesicles^19,21^. Zeta potential is a detection index of electric charge on the particle surface. In vivo, it can influence the distribution of niosomes, while it is thought that in vitro it might contribute to the physical stability of niosomes by reducing the rate of aggregation and fusion^15^. The addition of chitosan can significantly mitigate the negative properties of Nio-UA due to the electrostatic interaction between the positive charge on chitosan and the negative charge on UA^15,21^. Surface charge has been reported as affecting in vivo drug distribution. Several studies have revealed that positively charged nanoparticles show higher phagocytic and cellular uptake than negatively, neutrally charged, and PEGylated nanoparticles^22,23^. The positively charged nanoparticle will be endocytosized through clathrin receptors, while the negatively charged nanoparticles are primarily internalized via caveolin receptors^23^. However, other research into the bioavailability studies of nanoparticles has indicated that their negative charge increases the macrophage uptake more significantly than that of positively charged nanoparticles, thereby potentially reducing the effectiveness of nanodrug delivery^24^. Opsonin serum protein binding with negatively charged nanoparticles seems to occur to a higher degree than that of positively charged nanoparticles. Consequently, negatively charged nanoparticles are covered more extensively by opsonin proteins with greater stimulation of the phagocytosis by macrophages^25^.

Data on the weight of each organ indicated a reduced mean relative weight of the liver in the members of the four NDEA-induced groups compared to those of the normal group. Induction of NDEA causes hepatic degeneration that generally reflects loss of function associated with hepatocellular atrophy and injury^18^. A significant difference in relative liver weight occurred in the normal group compared to the UA and Nio-UA suspensions. In previous in vivo studies, administration of UA was known to reduce liver weight. UA can effectively relieve hepatic steatosis and reduce adipocyte size in the epididymis and decrease total cholesterol and triglycerides in the liver and plasma of subjects^26,27^. In this study, NDEA-induced subjects did not present a difference in relative spleen weight compared to members of the normal group.

NDEA is a well-known carcinogen that induces cancer of various organs in experimental animal subjects. Inducing liver cancer, NDEA can also result in lung adenocarcinoma^28^. Moreover, positively charged nanoparticles are also more easily taken up by lung cells, compared to neutral or negatively charged nanoparticles with the result that they can accumulate extensively in the lungs^29^. This may underlie the significant differences in the pulmonary organs, while in the heart, no changes were observed possibly due to differences in cell types and characteristics. However, further analysis of these organs is required.

The SGOT and SGPT levels in serum in the negative control group were recorded as higher than that in normal group. This indicates that the administration of NDEA 25 mg/kgBW to negative control group members on four occasions caused liver damage characterized by increased levels of SGOT and SGPT in blood serum. SGOT and SGPT are enzymes sensitive to liver cell damage which are predominantly contained in liver cells and, to a lesser extent, in muscle cells. Exposure to toxic substances causes a change in the permeability of the liver cell membrane resulting in damage or leakage, as a result of which the liver cells will release the enzymes they contain into the blood circulation, thereby increasing the levels of SGOT and SGPT and signaling liver disease^30^.

The levels of SGOT and SGPT in the negative control group were also higher than those in the Nio-UA and Nio-UA-CS groups. SGOT levels showed a significant difference (P < 0.05) while SGPT levels did not demonstrate a significant difference (P > 0.05) in the Nio-AU and Nio-UA-CS groups compared to the negative control group. This indicates that the administration of Nio-UA and Nio-UA-CS produces a hepatoprotective effect by reducing the release of SGOT and SGPT into the blood compared to UA suspension. A previous study of in vivo test results relating to paclitaxel niosomes indicated that the plasma drug concentration was higher in the paclitaxel niosome group than in the paclitaxel suspension group^31^. Oral use of niosomes can improve permeation and bioavailability, solubility of hydrophobic drugs, drug accumulation in the liver and controlled and targeted drug release^32^.

The SGOT level in the Nio-UA-CS group was lower than that of the Nio-UA group. The presence of chitosan can induce a greater effect marked by the release of fewer SGOT enzymes. This finding supports those of previous studies regarding the modification of UA liposomes with chitosan coating increasing bioavailability, slowing drug release in tumor tissue, and reducing dosage and potential side effects. This can happen because chitosan opens tight junctions in epithelial cells and allows drug to pass freely through epithelial cells via paracellular pathways^15^. Chitosan also induces mucosal adhesion through ionic interactions between positively charged amino groups and negatively charged functional groups on the surface of epithelial cells, thereby providing controlled release and absorption in the gastrointestinal tract^16^. Chitosan has good mucoadhesive properties that can prolong the residence time of the drug in the gastrointestinal tract. Under acidic conditions, chitosan will trigger the opening of tight junctions between epithelial cells and facilitate paracellular transport of niosomes^15^. Therefore, the nanoparticle system in the presence of chitosan coating can effectively improve oral absorption. There is still no information regarding the effect of chitosan on tight junctions in hepatocytes.

The levels of SGOT and SGPT in the UA suspension group were higher than in the negative control group, although they did not differ significantly. This is possible because the dose of 11 mg UA/kgBW administered is less effective if in the form of a suspension. The use of niosomes can overcome the problem of low drug solubility in water, thereby reducing drug dosage^33^. Previous research into the use of UA in the prevention of liver fibrosis due to CCl_4_ induction found optimal protection through the administration of UA at a dose of 50 mg/kgBW in distilled water containing 0.1% Tween 80^10,34^. Moreover, this is feasible due to the difference in the amount of UA taken because the UA suspension is insoluble. Consequently, there is a possibility that the preparation is not homogeneous, while the niosomes are more evenly dispersed than the suspension.

An analysis of the study results confirmed that the levels of SGOT and SGPT parameters in the Nio-UA and Nio-UA-CS groups were lower than in the normal group, although not significantly different. The lower the level, the healthier the condition of the liver^35^. In terms of further research, if experimental subjects are used, it is preferable to complete a sampling to check the levels of SGOT and SGPT before the subjects are treated to ensure that their initial condition is healthy.

It is evident from these observations that the administration of Nio-UA-CS can reduce inflammation, pleomorphism, dysplasia, and enlargement of hepatocyte cell nuclei in mice liver. These results indicate that the administration of chitosan to UA niosomes increases the anti-inflammatory and anticancer activity of UA^11^. This finding is consistent with those of previous studies regarding CS modification of liposomes which resulted in increased drug activity of UA liposomes and enhanced antitumor drug efficacy^15^. Liver histopathology observations were linear with the results of SGOT and SGPT levels indicating that the optimum repair of liver damage occurred in the Nio-UA-CS group followed by Nio-UA and, finally, UA suspension.

Spleen histopathology was also observed in the course of this study. Conventional nanoparticles are known to be trapped by RES, most of which will migrate to the liver and spleen^36^. Liposomes and lipid nanocarriers larger than 100–150 nm can be taken up by phagocytes. Monocytes, macrophages and neutrophils are phagocytes. The majority of these phagocytes reside in the liver and spleen for subsequent elimination^20^.

The administration of Nio-UA-CS indicates lymphoid tissue activation. Such activation is correlated with an increase in immune system activity^37^ which can protect the body from non-self-pathogens or cancer cells by destroying them^38^. In a previous study on UA nanoparticles with chitosan coating as folate-targeting, the preparation was shown to enhance tumor inhibition and promote an immune-boosting more effectively than free UA^39,40^.

It has been reported that Chitosan induces transient tight junction opening by translocating the membrane’s tight junction protein claudin-4 (Cldn4) into the cytoskeleton followed by its degradation in lysosomes^41,42^. Cldn4 has been recognised as a protein responsible for cell adhesion, polarity and paracellular permeability^43^. Intracelullar redistribution results in the weaking of the tight junction leading to the opening of the cells^41,42^. On the other hand, it has been reported that Cldn4 is not expressed in normal hepatocytes. However, its expression is increased due to fibrosis, rather than inflammatory condition, of severe liver injury^44^, which this gene expression correlates with differentiation of progenitor cells into mature hepatocytes. This study also reported that its expression was not found in cases of hepatocellular carcinoma. Therefore, chitosan’s effects on hepatocyte permeability and the drug’s penetration into deeper damaged liver tissue are still questionable, need to be further explored. In addition, NDEA induction has been reported to increase serum bilirubin levels^45^, and UA effectively reduced them, proving its potential efficacy for liver protection and promoting bile secretion^46,47^; however, this study was limited. Therefore, evaluating the serum bilirubin levels is vital to provide the information associated with the repair of liver damage and its dysfunctions^48^.

Chitosan coating on UA niosomes can improve the physical morphology of the liver, resulting in the relative weight of the liver and lung organs which are relatively the same as the normal group and there is no significant difference in the difference in body weight. Chitosan coating on UA niosomes can increase the effectiveness of UA as a therapy to prevent liver damage in subjects induced by N-Nitrosodiethylamine in terms of histopathological parameters of liver tissue which are relatively more normal than negative controls. Chitosan coating on UA niosomes can increase the effectiveness of UA as a therapy to prevent liver damage in mice induced by N-Nitrosodiethylamine in terms of decreasing serum levels of SGOT and SGPT.

## Methods

### Preparation of UA niosomes

Preparation of niosomes was conducted using a thin layer hydration method with a formula composition referred to previous studies as shown in Table 3^17^. UA (sigma-Aldrich, Tokyo, Japan) solution in methanol, span 60 (Wako Pure Chemical Industries, Ltd., Osaka, Japan), and cholesterol (Wako Pure Chemical Industries, Ltd., Osaka, Japan) in chloroform (Merck, Darmstadt, Germany) were mixed in a round bottom flask. The organic solvents were then heated in a rotary vacuum evaporator at a temperature of 60 °C until they had all evaporated and a thin lipid layer was formed. This layer was hydrated using 2 ml PBS solution pH 7.4 at 60 °C for 1 h^17^. Sonication was carried out with a water bath sonicator to form niosomes in order to reduce the size of the vesicles. Dissolving chitosan (Biotech, Cirebon, Indonesia) in 0.1 M acetic acid produced 0.1% chitosan solution which was subsequently diluted using distilled water to obtain a solution of 0.005% v/v chitosan which was added to the UA niosomal suspension. The addition was completed by mixing 40 µl of chitosan solution with 400 µl of niosomal samples before vortexing for ten seconds.

**Table 3:** Ursolic acid niosome formulation.

Formulation	Component (mol ratio)			Chitosan
	Span 60	Cholesterol	UA	
Nio-UA	60	40	10	−
Nio-UA-CS	60	40	10	+

*UA* ursolic acid, *CS* chitosan, ( −) without chitosan addition, ( +) with chitosan addition.

### Physical characterizations of UA niosomes

Approximately 100 µL niosomes was diluted in 2 mL aqua demineralization with particle size and PDI measurements subsequently being completed by the Dynamic Light Scattering method using Malvern Zetasizer Instruments (Malvern Panalytical, UK). Furthermore, 100 µL niosomes were also taken diluted in 2 mL aqua demineralization ζ-potential measured using the Electrophoresis Light Scattering method with Malvern Zetasizer Instruments (Malvern Panalytical, UK). The evaluation was completed three times for each of the Nio-UA and Nio-UA-CS samples.

### In vivo efficacy evaluation of UA niosomes in mice induced with NDEA

The use of experimental animals in this research was approved following an ethical feasibility test conducted on April 1, 2022 at the Faculty of Veterinary Medicine, Universitas Airlangga by the Faculty’s Research Ethics Commission through the issuance of Certificate of Ethics Eligibility No. 2.KEH.035.04.2022**.** All methods were performed in accordance with ARRIVE guidelines and relevant regulations^49^. In this study, 6-week-old male mice (*Mus musculus*) Balb/c represented the subjects. Determination of the number of sample replications employed the Federer’s Formula. Five randomly selected subjects formed the members of each treatment group. The negative control group was treated by means of NDEA i.p. injection for four weeks, while PBS pH 7.4 was administered orally during sample treatment.

### Induction of liver damage of mice by NDEA injection

Induction of liver damage in subjects was achieved through the intraperitoneal administering of a 25 mg/kgBW dose of NDEA (sigma-Aldrich, Tokyo, Japan)^50^ once a week for four weeks. Evaluation of the resulting liver damage was effected by recording the subjects’ body weight on a weekly basis during the test period to identify any increase or decrease.

### Administration of UA niosomes into mice induced with NDEA

Subjects were given drugs, including UA suspension in 0.5% CMC Na, Nio-UA, and Nio-UA-CS, according to whichever group they belonged. The UA dose was equivalent to 11 mg UA/kgBW^40^. The drug was administered orally using a needle probe seven and three days before NDEA induction and was continued once a week together the intraperitoneal induction of NDEA at a dose of 25 mg/kgBW for the subsequent four weeks.

### SGOT and SGPT evaluation of mice induce with NDEA after administration of UA niosomes

After the final UA preparation had been administered, the subjects were left for seven days before their organs were surgically removed. Having been given intraperitoneal anesthesia in the form of a 10 mg/kgBW dose of ketamine, a blood sample was taken from the inferior vena cava, inserted into test tubes and centrifuged at 6000*g* × force for 15 min at 4 °C to obtain serum whose levels of SGOT and SGPT was then determined using the International Federation of Clinical Chemistry and Laboratory Medicine (IFCC) 37 method. The decrease in SGOT and SGPT levels was determined from comparisons between each treatment group and the control group. The SGOT and SGPT levels were determined by enzymatic reaction kinetic method. The reagents used were ready-to-use reagents consisting of AST (GOT) and ALT (GPT) reagents^51^.

### Histopathological evaluation of liver and spleen of mice induce with NDEA after administration of UA niosomes

Following extraction of the blood sample, the subjects’ spines were dislocated. The subjects were dissected and their livers immediately removed, rinsed with normal saline, and dry wiped with a tissue or filter paper, before finally being weighed, photographed and morphologically examined. The liver sections were fixed in 10% neutral buffered formalin and then stained with haematoxylin and eosin (H&E staining) for further histological analysis of the differences in appearance between the livers of the normal and treated subjects^11^. Changes in lobular architecture, bleeding, neutrophilic infiltration, and dysplastic hepatocytes on histopathological preparations of liver tissue were observed by means of light microscopy^45,52^. To evaluate the organ weight of the subjects, quantitatively each organ of mice in each group was weighed. Because overall body weight affects the weight of individual organs, the relative weight of the livers was calculated using the formula^53^:

$$\mathrm{Relative\, Weight }= \frac{Absolute\, organ\, weight (g)}{Body\, Weight (g)} \times 100\%.$$

The calculation results relating to the relative weight of the organs in the treatment group were then compared with those of the normal and negative control groups to determine whether significant differences existed.

### Statistical analysis

The quantitative data represent the average and standard deviation of sample measured in replications. A statistical analysis was performed using the one-way variant analysis (ANOVA) method followed by a Post Hoc Tukey HSD test. The *P value* < 0.05 is considered as a significant difference between the results.

### Ethical conduct of research statement

The animal study procedures were performed in accordance with the ethical clearance issued by The Ethics Commission of Faculty of Veterinary Medicine, Universitas Airlangga (Certificate number 2.KEH.035.04.2022 dated April 1, 2022).

## Data Availability

The datasets used and/or analysed during the current study available from the corresponding author on reasonable request.
